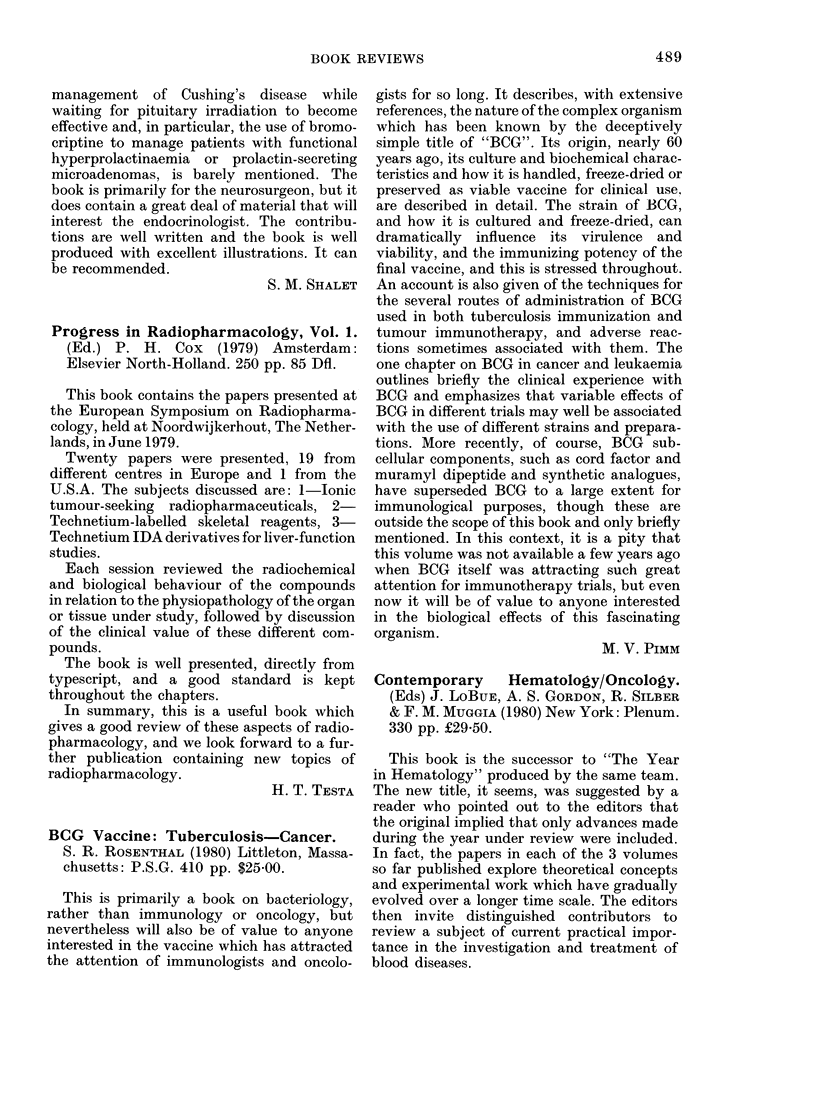# BCG Vaccine: Tuberculosis—Cancer

**Published:** 1980-09

**Authors:** M. V. Pimm


**BCG Vaccine: Tuberculosis—Cancer.** S. R. ROSENTHAL (1980) Littleton, Massachusetts: P.S.G. 410 pp. $25·00.

This is primarily a book on bacteriology, rather than immunology or oncology, but nevertheless will also be of value to anyone interested in the vaccine which has attracted the attention of immunologists and oncologists for so long. It describes, with extensive references, the nature of the complex organism which has been known by the deceptively simple title of "BCG". Its origin, nearly 60 years ago, its culture and biochemical characteristics and how it is handled, freeze-dried or preserved as viable vaccine for clinical use, are described in detail. The strain of BCG, and how it is cultured and freeze-dried, can dramatically influence its virulence and viability, and the immunizing potency of the final vaccine, and this is stressed throughout. An account is also given of the techniques for the several routes of administration of BCG used in both tuberculosis immunization and tumour immunotherapy, and adverse reactions sometimes associated with them. The one chapter on BCG in cancer and leukaemia outlines briefly the clinical experience with BCG and emphasizes that variable effects of BCG in different trials may well be associated with the use of different strains and preparations. More recently, of course, BCG subcellular components, such as cord factor and muramyl dipeptide and synthetic analogues, have superseded BCG to a large extent for immunological purposes, though these are outside the scope of this book and only briefly mentioned. In this context, it is a pity that this volume was not available a few years ago when BCG itself was attracting such great attention for immunotherapy trials, but even now it will be of value to anyone interested in the biological effects of this fascinating organism.

M. V. PIMM